# POSS Nanofiller-Induced Enhancement of the Thermomechanical Properties in a Fluoroelastomer Terpolymer

**DOI:** 10.3390/ma11081358

**Published:** 2018-08-06

**Authors:** Daphné Berthier, Marie-Pierre Deffarges, Nicolas Berton, Mathieu Venin, Florian Lacroix, Bruno Schmaltz, Yohan Tendron, Eric Pestel, François Tran-Van, Stéphane Méo

**Affiliations:** 1 Laboratoire de Mécanique Gabriel Lamé, Université de Tours, Université d’Orléans, INSA Centre Val de Loire, Polytech Tours, 7 avenue Marcel Dassault BP40, 37004 Tours, France; marie-pierre.deffarges@univ-tours.fr (M.-P.D.); mathieu.venin@univ-tours.fr (M.V.); florian.lacroix@univ-tours.fr (F.L.); stephane.meo@univ-tours.fr (S.M.); 2 Laboratoire PCM2E (Physico-Chimie des Matériaux et des Electrolytes pour l’Energie), Université de Tours, Parc de Grandmont, 37200 Tours, France; nicolas.berton@univ-tours.fr (N.B.); bruno.schmaltz@univ-tours.fr (B.S.); francois.tran@univ-tours.fr (F.T.-V.); 3 Zodiac Aerosafety Systems, 20 avenue Georges Pompidou, 37600 Loches, France; yohan.tendron@zodiacaerospace.com (Y.T.); eric.pestel@zodiacaerospace.com (E.P.)

**Keywords:** POSS, nanofillers, FKM, rubber, terpolymer, thermomechanical properties

## Abstract

The present study reports on the use of three types of polyhedral oligomeric silsesquioxanes (POSS) nanoparticles with various organic substituents as fillers in a fluoroelastomer (FKM). A series of/POSS elastomer composite thin films is prepared. Microstructural SEM/TEM (scanning electron microscopy/transmission electron microscopy) imaging reveals a dispersion state allowing the presence of micron-sized domains. The influence of POSS content is studied in order to optimize thermal stability and mechanical properties of the composite thin films. Both POSS-A (with an acryloyl functional group and seven isobutyl substituents) and POSS-P (with eight phenyl substituents) lead to higher thermal stability and modulus of the composites, with respect to the unfilled FKM terpolymer matrix. covalent grafting of POSS-A onto the FKM network is found to play a critical role. Enhanced storage modulus in the rubbery plateau region (+210% at 200 °C for 20 phr) suggests that POSS-A is particularly suitable for high temperature applications.

## 1. Introduction

Since their first use, fluoroelastomers (FKM) have become of great interest. They are used for many applications in the building (as paints and coatings resistant to UV and graffiti), petrochemical and automotive industries, aerospace, and aeronautics (use of elastomers as seal, gasket or O-ring to be used in extreme temperatures for liquid hydrogen storage in space shuttles), chemical engineering (high-performance membranes), optics (core and cladding of optical fibers), and treatment of textiles [1]. This polymeric family includes different kinds of products with specific properties, such as thermoplastics and elastomers [1]. Fluorinated FKM terpolymers consisting of vinylidene fluoride (VDF), hexafluoropropylene (HFP), and tetrafluoroethylene (TFE) monomer units can be either amorphous or semi-crystalline depending upon the monomers ratio [2]. Their specificity consists of a unique combination of properties including good thermal aging, weather, and chemical resistances, low surface energy, low inflammability, and low moisture absorption [3,4]. These properties can be explained by a low polarizability and a strong electronegativity provided by fluorine atoms leading to low molecular motions and high bond energy [5].

However, improving the resistance of FKM based terpolymer is still required as they exhibit chemical degradation as well as thermal degradation at high temperature in demanding environment. This involves oxidation and thermolysis of the polymer that can occur on vinylidenefluoride groups and cross-linking bonds [6,7]. A way to further improve thermal properties of FKM is to integrate nanoparticles. Indeed, this general process seems to be the simplest mean to enhance various rubber characteristics, including thermal resistance [8]. A major advantage of nanocomposites is that a low fillers content is sufficient to improve their properties [9,10]. The tensile resistance of natural rubbers is for instance found to increase from around 5 MPa with carbon black (10 phr: per hundred rubber), up to 15 MPa with nanoclay (10 phr) [11]. In particular, the presence of nanofillers was found to lead to higher cross-linking density upon vulcanization.

Polyhedral oligomeric silsesquioxanes (POSS) nanoparticles are particularly attractive as nanofillers. They were first developed by the United States Air Force for aerospace applications [12]. Scott [13] isolated the first (CH_3_SiO_1.5_)_n_ oligomers in 1946, with other volatile compounds, by thermolysis of polymer products obtained from methyltrichlorosilane and silane dimethylchloro-cohydrolysis. POSS is described as a cubic cage with a silicon-to-oxygen ratio of 1:1.5, organic peripheral groups at each silicon atom [14,15], and a molecular diameter of around 1–3 nm [8]. The number and nature of functional groups present on the POSS corners lead to different kinds of interactions with the matrix [16,17]. These nanoparticles are very adaptable and can be fully designed to the convenience of the user. In the absence of functional groups, POSS behave as a classical filler interacting with the matrix through physical interactions. On the other hand, POSS can be chemically grafted as pendant units on macromolecular chains with functional groups on its periphery [18]. When two functional groups are present, POSS can be used as a cross-linking agent leading to tridimensional networks. Currently, incorporation of POSS into polymers increases the oxidative resistance, the glass transition temperature, and the composite stiffness. Various types of POSS have been used with PDMS [19,20,21,22,23,24] or natural rubber matrix [25]. Despite few promising findings, applications with a FKM matrix are much less well described. The amount of fillers required to obtain an efficient level of reinforcement is likely to depend on the mixture and the application. A 48% increase of elongation at break and 93% increase of the tensile at break were reported, with only 5 phr of POSS functionalized with hydroxyl functional groups [26]. In another work, a mixture with 12 phr (per hundred rubber) of POSS with propyl methacrylate on its periphery, resulted in an increase by 10% of the temperature at 2% of weight loss measured by thermogravimetric analysis (TGA) [27].

Here, three kinds of POSS nanoparticles, differing by their organic substituents, are used as nanofillers in a (HFP–TFE–VDF) fluoroelastomer terpolymer. The influence of specific POSS/FKM interactions on the FKM network structure and the thermal stability of the composites is studied for each type of POSS. The most appropriate fillers are selected and the amount is optimized in order to enhance the thermomechanical properties of the fluoroelastomer composites. The covalent grafting of POSS onto the fluoroelastomer network is found to lead to significant improvement.

## 2. Materials and Methods 

### 2.1. Materials

The matrix was a FKM terpolymer (HFP–TFE–VDF) provided by DuPont© (Wilmington, DE, USA), and it contained a tiny amount of barium sulfate. The crosslinking system was formed by an organic peroxide coupled with a crosslinking coagent. The peroxide was a diacyl peroxide Norperox DBPH-45 (NODMANN, RASSMANN GMBH, Hamburg, Germany), with silicone carbonate as phlegmatizer (2,5-Dimethyl-2,5-di-(ter-butylperoxy)hexane). The coagent was used to enhance the crosslinking process, the heat resistance and mechanical properties, with calcium carbonate as a phlegmatizer (triallylisocyanurate Norlink TAIC 70 CS, (NODMANN, RASSMANN GMBH, Hamburg, Germany) Three types of POSS (RSiO_1.5_)_n_ were used: POSS-P Octaphenyl, POSS-F Trifluoropropyl and POSS-A Acryloisobutyl provided by Hybrid Plastics (Hattiesburg, MS, USA). POSS-F has a T_12_ cage structure (bearing 12 trifluoropropyl moieties (CF_3_-)), POSS-P possesses a T_8_ structure (with eight phenyl moieties (C_6_H_5_-)) and POSS-A has a T_8_ structure (with seven isobutyl moieties (C_4_H_9_-) and an acrylo functional group (-C_6_O_2_H_10_)). The three POSS have the appearance of a white powder.

### 2.2. Compounding and Vulcanization Procedure

The nanocomposites were prepared by solution casting (Hand Coater). The ingredients were dispersed in acetone by magnetic stirring at room temperature in the following integration order: POSS, crosslinking system, and finally the matrix. The matrix solution concentration is 383 g/L. For each POSS, five loadings were studied: 5, 10, 15, 20, and 40 phr (per hundred rubber).

The composite designation was: -F for the FKM gum; -F_R_ for the mixture of FKM gum and the crosslinking system; -F_T_ for the mixture of gum, the crosslinking system, and POSS fillers, following by the POSS type (A, F, or P) and the rate. For example: the F_T_ composite with POSS-A at 15 phr was called: F_TA-15_.

Rubber elaboration was carried out in thin films (350–400 µm), processing from solution using a Hand Coater (Elcometer 4340, elcometer®, Manchester, UK) and dried for 13 days at room temperature [28]. One should note that only crosslinker containing samples were cured. During vulcanization, the samples were heated for 45 min at 150 °C, followed by post-curing at 180 °C for six hours, under an air atmosphere.

### 2.3. Characterization

A Zeiss field emission model Ultra Plus Scanning Electron Microscope (SEM) (Carl Zeiss Microscopy, Oberkochen, Germany), operating at 5 kV, was used to examine the nanocomposites surface micro-structure and analyze particle dispersion. The samples are cleaved with a sharp razor and coated with a Platinum film to increase electrical conductivity. A Transmission Electron Microscopy (TEM) (JEOL 1230, 120kV, JEOL USA, Inc., Peabody, MA, USA) was used to observe the particle dispersion inside the nanocomposite. The nanoparticles were dispersed in acetone, drop-casted on carbon coated copper grid, and were analyzed under an accelerating electron beam, after solvent evaporation. Cured nanocomposites were ultramicrometered using a diamond knife at −40 °C with a Leica Ultracut UCT (Leica Microsystemes SAS, Nanterre, France). These ultra-thin sections were then transferred onto a copper grid covered with a very thin film of amorphous carbon.

Differential scanning calorimetry (DSC) was performed under a nitrogen atmosphere on a Netzch Instruments (DSC 200 F3, Netzch, Selb, Germany). The accuracy of the measures was ±1 °C. Samples are cooled down to −140 °C and heated up to 250 °C at 20 °C·min^−1^. A second cooling was performed from −80 °C to 250 °C at 20 °C·min^−1^. Systematically, two DSC measurements have been performed on each samples. The averaged values of glass transition temperature (T_g_) and of cross-linking reaction enthalpy (ΔH) were reported. Thermal gravimetric analysis (TGA) is performed using a Mettler–Toledo instrument (TGA-2, Mettler–Toledo, Columbus, IN, USA). Samples are characterized under two different atmospheres. For the first procedure, samples were heated from 25 °C to 600 °C under nitrogen atmosphere, then from 600 °C to 800 °C under air, at 20 °C·min^−1^. For the second procedure, samples are heated from 25 °C to 800 °C under air at 20 °C·min^−1^. T_onset_ (extrapolated starting temperature, intersection, point of the baseline before a thermal effect and tangent), T_5%_, T_20%_ (temperature for 5 and 20% of weight loss) and T_50%_ (temperature of peak decomposition of the derivative curve) are measured. Theoretical ashes are calculated assuming a complete conversion of POSS to SiO_2_. The residues (in percent) calculated from each thermogram are also given. These residues correspond to ash content remaining after measurement. An average of three independent measurements was reported.

The dynamic mechanical properties were analyzed with a thermal analysis instrument (DMA 2980 TA Instrument Dynamic Mechanical Analyzer; TA Instruments, New Castle, PA, USA). Test specimens were taken from the post-cured thin film (width 9 mm, distance between clamping 10 mm) and were submitted to a tension mode. Temperature sweeps were carried out over a temperature range from −30 °C to 250 °C (3 °C·min^−1^) at a constant frequency (1 Hz) and with an amplitude of displacement of ±0.5 µm. An average value of three independent measurements was reported: the glass transition temperature T_g_ taken at the maximum phase angle between stress and strain (tan δ), tan δ at (T = T_g_), and the storage modulus (E’) at 100 °C and at 200 °C.

**Figure 1:**
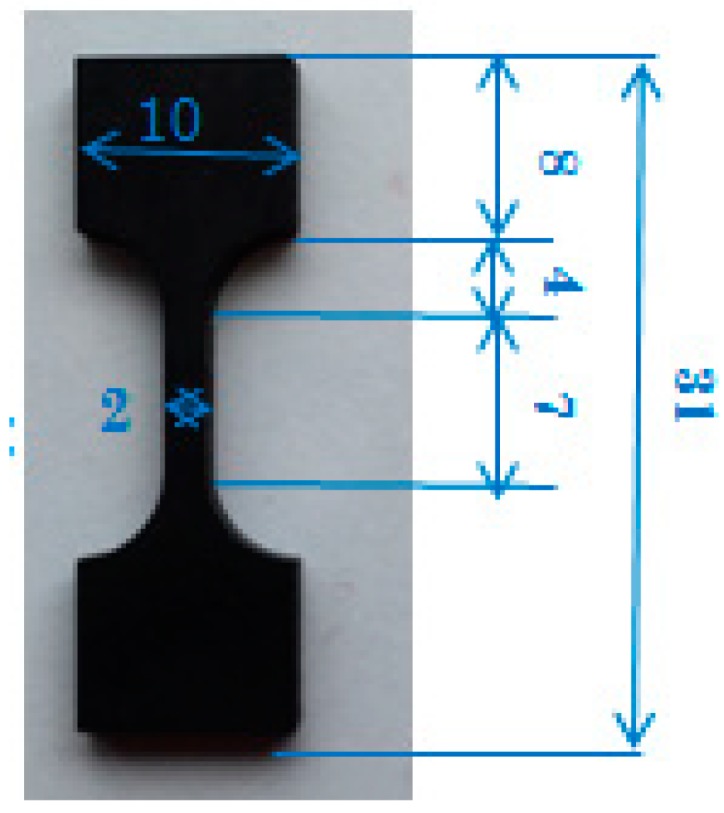
Dumbbell-shaped specimens developed in our laboratory.

A universal testing machine (Zwick 1kN; Zwick Roell Group, Ulm, Germany) with computerized recording was used at room temperature to obtain the mechanical properties. The tests were carried out at a crosshead speed of 1 mm/min (or έ = 0.083%/s), and an average of at least 10 measurements for each compound was recorded. The test dumbbell-shaped specimens were taken from the post-cured thin film with a specific tensile specimen cutter developed in our laboratory, in order to optimize the mechanical response of the thin film on the test machine, and to enlarge the clamping area of the dumbbell-shaped specimens. The useful length and the width of dumbbell-shaped specimens is respectively 15 mm and 2 mm (Figure 1). The averaged values of tensile at break, elongation at break, and moduli at 10% and at 100% of deformation are reported.

## 3. Results and Discussion

### 3.1. Composition and Fabrication of Composite Films

**Scheme 1:**
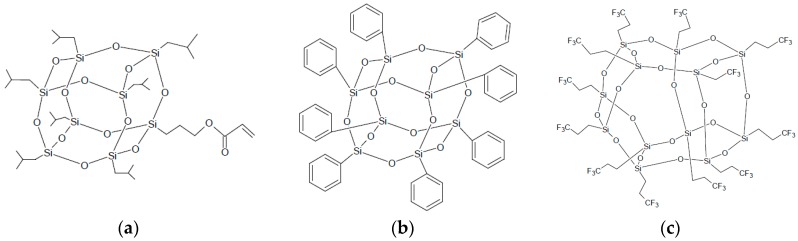
Chemical structure of: (**a**) POSS-A; (**b**) POSS-P and (**c**) POSS-F.

The chemical structure of the FKM was composed of three monomers: HFP (CF_2_CF(CF_3_)), TFE (CF_2_CF_2_) and VDF (CH_2_CF_2_). The various POSS investigated in this study are shown in Scheme 1. Specific interactions with the FKM could be expected for each type of POSS. POSS-A was likely to be covalently grafted on the FKM due to the presence of a reactive acryloyl functional group on its surface, while POSS-P and POSS-F only led to physical interactions of different strengths. In particular, the presence of fluorinated moieties on POSS-F provided improved chemical compatibility with the (HFP–TFE–VDF) terpolymer. The POSS-containing nanocomposites based on FKM were obtained as thin films (350−400 µm thick) using doctor blade coating. The vulcanization process was then performed, as described in the experimental section.

### 3.2. Evaluation of the Dispersion State

The distribution of fillers can influence the properties of the final products. Microstructural analysis was performed using transmission (TEM) and scanning (SEM) electron microscopy in order to determine the particles size, dispersion state, and the morphology of the nanocomposites.

**Figure 2:**
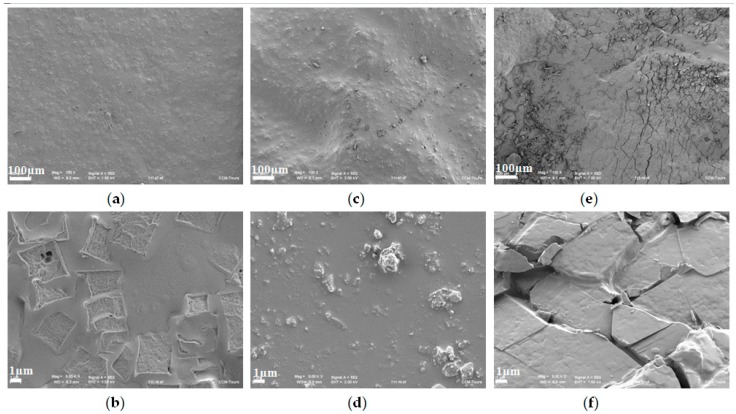
Scanning electron microscopy (SEM) micrographs of POSS nanocomposites. (**a**,**b**) F_TA-20_; (**c**,**d**) F_TP-20_; (**e**,**f**) F_TF-20_ (POSS content 20 phr).

SEM micrographs depict the surface of the three nanocomposite films (Figure 2). SEM images of the FKM polymer matrix F (Figure S1), and of the vulcanized matrix F_R_ (Figure S2) are shown for comparison. The composites obtained with POSS-A (F_TA_) and POSS-P (F_TP_) exhibited a smooth surface morphology, whereas F_TF_ nanocomposite films presented a heterogeneous surface, with cracked and lamellar zones. This can be attributed to low filler/matrix compatibility. In F_TA_ and F_TP_ composites, the charges seemed well dispersed, indicating a better compatibility with the FKM. The typical micrometer-sized POSS-A crystals with rectangular faces were observed on TEM images (Figure S3) are visible at the surface of the F_TA_ film (Figure 2b), while, crystalline phases are not evidenced from SEM images in F_TP_ and F_TF_ films (Figure 2d,f).

**Figure 3:**
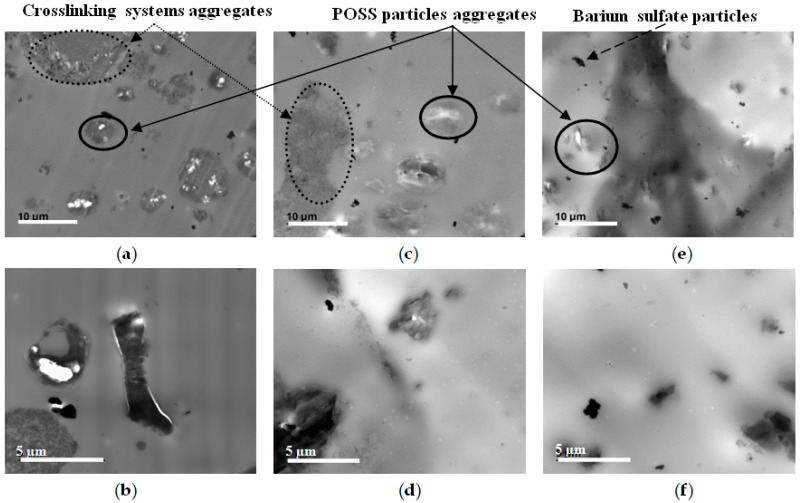
Transmission electron microscopy (TEM) micrographs of POSS nanocomposites. (**a**,**b**) F_TA-20_, (**c**,**d**) F_TP-20_, (**e**,**f**) F_TF-20_ (POSS content 20 phr).

In order to investigate the fillers dispersion within the matrix, TEM micrographs were recorded on ultra-thin sections of the nanocomposite films containing POSS-A, POSS-P, or POSS-F at a loading ratio of 10 phr (Figure 3). The observed small dark particles came from the FKM polymer gum (Figure S4), and large bright domains (>10 µm) could be attributed to crosslinking system aggregates dispersed in the matrix (Figure S5). To identify these particles, energy dispersive X-ray spectrometry (EDX, Carl Zeiss Microscopy, Oberkochen, Germany) studies were performed. In the black particles zones, sulfur, oxygen, and barium chemical elements were detected (Figure S6) and thus may correspond to barium sulfate. This ingredient was added into the rubber matrix in order to avoid coalescence by agglomeration during the polymerization. In the aggregate zones, silicon and carbon elements were identified (Figure S7), thus confirming their assignment to crosslinking system compounds. Indeed, it was fixed on an inert support, including the detected elements. POSS particles aggregates exhibit both dark and bright spots, likely due to different crystal orientations. The POSS dispersion state within the bulk nanocomposites can be deemed acceptable since POSS aggregates are smaller than aggregates related to cross-linking agents (<10 µm).

### 3.3. Thermal Characterization

The thermal properties of the composites were characterized by DSC and TGA for different POSS loadings.

#### 3.3.1. DSC

**Figure 4:**
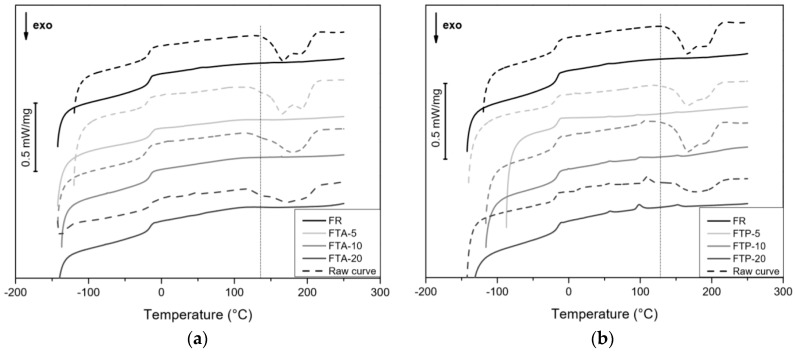

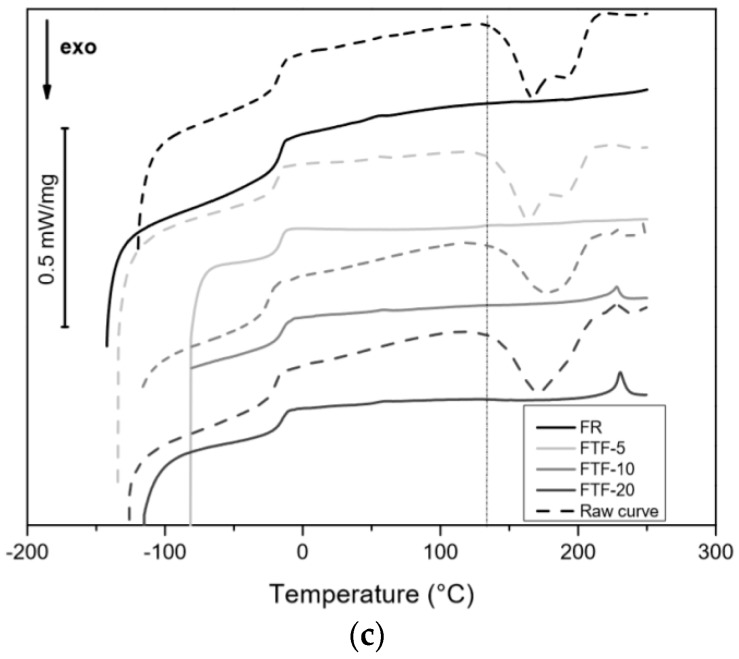
Differential Scanning Calorimetry (DSC) curves of raw and cured F_T_ nanocomposites: (**a**) F_TA_, (**b**) F_TP_, and (**c**) F_TF_ at a heating rate of 20 °C·min^−1^.

To understand the impact of POSS incorporation, DSC measurements were carried out on the raw composite mixtures, and on the vulcanized nanocomposites. A glass transition temperature (T_g_) was clearly observed in all cases (Figure 4). The exothermic peaks that were observed upon heating of the raw composite films could be attributed to the thermally activated cross-linking reaction. The corresponding heat of reaction (ΔH) was measured from its peak area. Data are summarized in Table 1.

POSS filling was found to have only a limited influence on the glass transition temperature. The glass transition of the unfilled FKM matrix F_R_ was −16.8 °C and it increased very slightly to −16.2 °C upon vulcanization. With the presence of POSS, the T_g_ of the raw composites was slightly lower, regardless of POSS surface functional group and content. This indicates that POSS particles acted mainly as plasticizers, bringing additional free volume. At a higher POSS content (>10 phr), it can be noticed that the T_g_ increased again with POSS-A and POSS-F, which may be related to a competing effect of POSS/FKM interactions [29]. However, these variations remained small compared with some previous reports where the T_g_ of POSS-containing polymers was found to be strongly dependent on the nature of surface functional groups present on POSS particles [27,30]. This suggests that intermolecular interactions between fillers and FKM were weak. Vulcanization of the composites led to a noticeable increase of T_g_ that canceled the plasticizing effect of the filler. The average T_g_ values measured on the vulcanized samples were all analogous to, or even slightly higher than the T_g_ of the FKM matrix. The accuracy of the measure (±1 °C) did not allow to a particular trend related to the POSS type or amount in the composite to be determined.

**Scheme 2:**
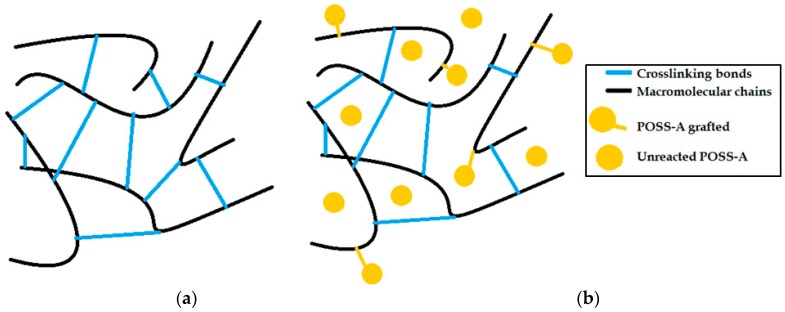
Schema of the crosslinked rubber: (**a**) without fillers and (**b**) with POSS-A as pendant groups.

The cross-linking reaction can be evaluated from the area under the exothermic peaks. The ΔH values recorded for F_TP_ compounds decreased when the filler content increased, indicating that POSS-P particles hamper the cross-linking reaction. It can also be noticed that the onset of the exothermic reaction peak was shifted toward higher temperature (*ca.* +10 °C at 20 phr loading) (Figure 4b). On the contrary, the vulcanization process showed little sensitivity to the presence of POSS-F (Figure 4c). The temperature at which cross-linking was initiated remained unchanged, and ΔH (in J/g of matrix) was close to those measured for the FKM matrix F_R_. The vulcanization of F_RA_ compounds exhibited a more complex behavior. Upon the incorporation of POSS-A, a shift towards low temperatures appeared in the crosslinking area on the thermograms in addition to the characteristic exothermic cross-linking peaks, and exothermic reactions started at a lower temperature (onset was shifted from 145 °C to 125 °C) (Figure 4a). This phenomenon could be associated with the grafting of POSS particles on the polymer chains through the reactive acryloyl groups. As a consequence, the heat of reaction was strongly enhanced at a loading of 5 phr. Fillers act as an inductive donor effect; the chains are more reactive and this could explain ΔH increase. However, it was noticed that ΔH decreases at a higher loading. This could be due to a steric hindrance induced by POSS-A particles that act as a shield because of the inductive attractor effect. There is most likely a competition between grafting and cross-linking reactions, as the grafted POSS particles reduce the number of available cross-linking sites on the FKM chains (Scheme 2). Indeed, as described in the general introduction, with one functional group, POSS-A can only be grafted onto the matrix. Furthermore, particles with unreacted double bonds could also remain in the matrix and/or potentially react together.

Grafting and cross-linking reactions are yet not straightforward to quantify, as the temperature range of those two phenomena seemed to overlap. Swelling tests were considered. With this technique, unreacted POSS could be released in the swelling solvent and could disturb the interpretation of the experiment by participating to the mass loss. Moreover, with this test, it would be difficult to distinguish the crosslinking reaction part to the grafting reaction.

Figure 4b,c present respectively endothermal peaks at around 50 °C, 100 °C, and 150 °C, and at ~225 °C. The intensity of these peaks increased with the increase of filler loading, and was attributed by DSC thermograms performed on the fillers alone, to the fusion of POSS crystal structure (Figure S8).

#### 3.3.2. TGA

**Figure 5:**
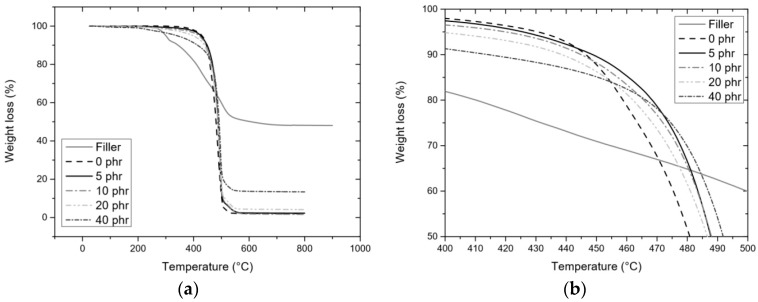
Thermal Gravimetric Analysis (TGA) curves of POSS-A filler and F_TA_ nanocomposites at different filler loading under air. (**a**) unzoomed; (**b**) zoomed.

**Figure 6:**
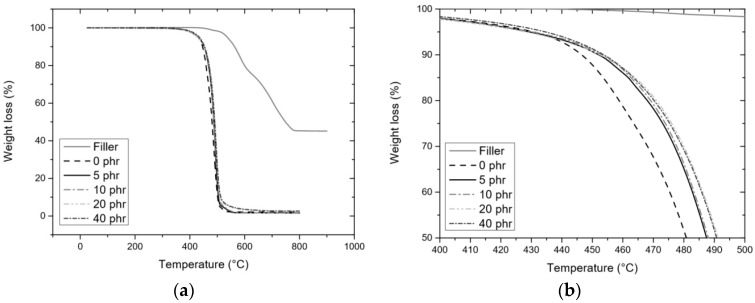
Thermal Gravimetric Analysis (TGA) curves of POSS-P filler and F_TP_ nanocomposites at different filler loading under air. (**a**) unzoomed; (**b**) zoomed.

**Figure 7:**
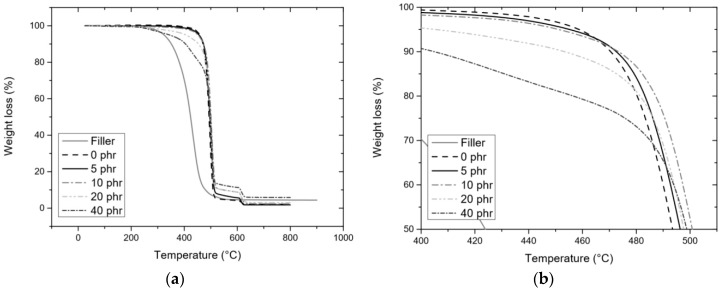
Thermal Gravimetric Analysis (TGA) curves of POSS-A filler and F_TA_ nanocomposites at different filler loading under nitrogen. (**a**) unzoomed; (**b**) zoomed.

**Figure 8:**
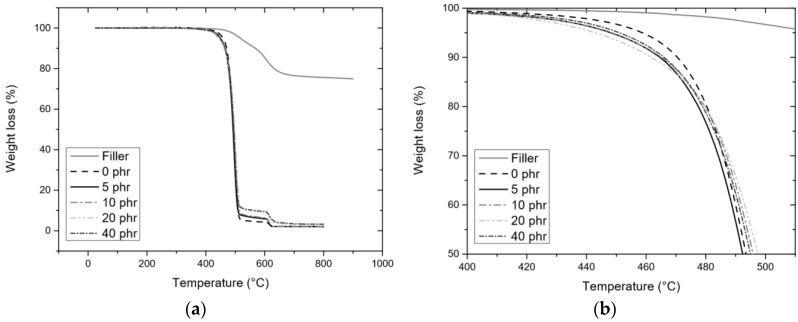
Thermal Gravimetric Analysis (TGA) curves of POSS-P filler and F_TP_ nanocomposites at different filler loading under nitrogen. (**a**) unzoomed; (**b**) zoomed.

**Figure 9:**
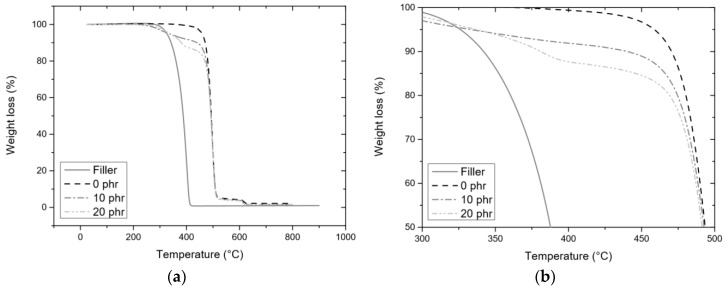
Thermal Gravimetric Analysis (TGA) curves of POSS-F filler and F_TF_ nanocomposites at different filler loading under nitrogen. (**a**) unzoomed; (**b**) zoomed.

The FKM polymer, POSS particles, and samples of composite system F_T_ modified with 5–40 phr of POSS were analyzed using TGA under oxidative atmosphere (Figure 5 and Figure 6), and under a non-oxidative (nitrogen) atmosphere (Figure 7, Figure 8 and Figure 9), in order to characterize the thermal stability of each system.

The fluorinated terpolymer matrix exhibits strong thermal stability. The FKM polymer F had a 5% decomposition temperature equal to 452 °C under nitrogen, and 398 °C under air (Figure S9). The addition of a crosslinking system (F_R_) followed by a vulcanization process increased the thermal properties by 6 °C under nitrogen, and by 32 °C under air (Figure S10). The TGA data summarized in Table 2 show that POSS incorporation in polymer matrix can either lead to considerable improvement or decrease of thermal stability as a function of POSS periphery. T_5%_ of the various composites was strongly influenced by the thermal stability of the POSS filler itself. Therefore, the temperature at maximum weight loss (*ca.* 50% weight loss in all cases) may provide a more appropriate assessment of thermal stability of the composites as it was likely to mostly reflect the degradation of the FKM network. T_onset_, which represents the extrapolated starting temperature, i.e., the beginning of the degradation process involved, is also a common indicator [31]. This criterion takes into consideration the starting decomposition and depends on the degradation speed.

Both POSS-A and POSS-F exhibited significant weight losses at lower temperatures than did the FKM matrix. The T_5%_ of F_TA_ and F_TF_ composites consequently decreased upon addition of POSS. While the thermal resistance of POSS-P was better, this did not translate into higher T_5%_ for F_TP_. Regardless of the atmosphere, POSS-P decomposed faster and completely in presence of the matrix once the FKM weight loss occurred (Figure 6 and Figure 8), which suggests the release of fluoride anions attacking the POSS silicon cage. The release of hydrofluoric acid upon the degradation of FKM is known to occur through dehydrofluorination, leading to the formation of double carbon bonds on the polymer backbone [7,32]. This was consistent with the char yield reduction. Indeed, POSS decompose ideally to silica form [33]. The theoretical ash (residue percentage) for the total conversion of the POSS cage was calculated (Table 2). The higher residue percentage obtained for POSS-P under nitrogen atmosphere (74.7%), with respect to inorganic Si–O fraction (ca. 40% wt.) can be attributed to the entrapment of carbon in the structure [34] (Figure 8). F_TP_ ashes are clearly lower than the theoretical ash, confirming the elimination of the formation of SiO_2_ residue [27].

Most importantly, it was noticed that both POSS-A and POSS-P led to higher T_onset_ and T_50%_ values. T_onset_ increases with the filler loading. F_TA_ exhibited better improvement under air than under nitrogen (+18 °C vs. +14 °C at 40 phr with respect to F_R_) (Figure 5 and Figure 7). Similarly, in regards of T_onset_, F_TP_ seemed to present better thermal resistance under air atmosphere (+10 °C at 20 phr under air vs. +2 °C at 40 phr under nitrogen), as observed with a polystyrene matrix in the literature [35]. This improvement under thermal oxidative environment could be linked to the formation of a silica (SiO_2_) layer on the surface, hence serving as a barrier and preventing further degradation of the matrix [36]. T_50%_ showed a similar trend, with values reaching 493 °C (+5 °C) and 502 °C (+5 °C) for F_TA_ under air and nitrogen, respectively. For F_TP_, T_50%_ up to 496 °C (+8 °C) and 501 °C (+4 °C) were determined under air and nitrogen, respectively. Thus, based on the assumption that T_onset_ and T_50%_ values provided insights into the degree of decomposition of the FKM network, it can be concluded that POSS-A and POSS-P fillers improved the thermal stability of the nanocomposites. At the same time, no significant enhancement was observed with POSS-F (Figure 9 and Table 2).

### 3.4. Mechanical Characterization

#### 3.4.1. Tensile Tests

**Figure 10:**
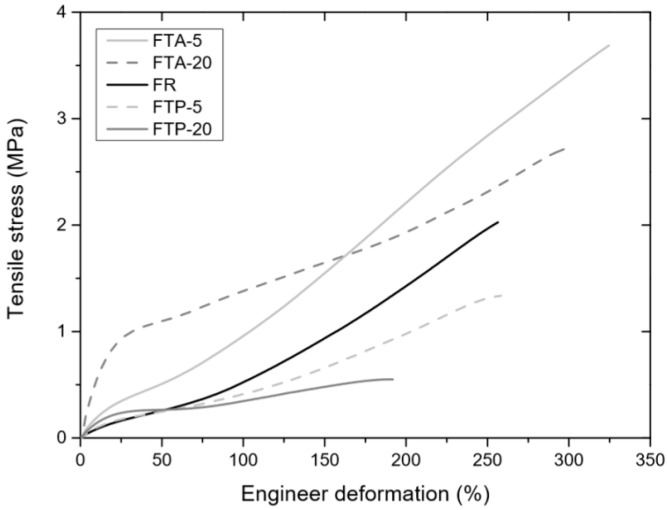
Stress-strain curves of F_TA_ and F_TP_ mixtures at 5 and 20 phr at room temperature.

Given appropriate film forming properties and promising thermal behavior, POSS-A and POSS-P composites were selected for further investigations of their mechanical properties. Measurements were realized without the elimination of Mullins effect [37]. The tensile properties of the compounds were measured at a cross-head speed of 1 mm/min (or έ = 0.083%/s) at room temperature (Figure 10). A low rate of deformation was fixed in order to remove the viscosity effect. The values of ultimate tensile stress, elongation at break, modulus at 10% elongation, and modulus at 100% elongation for the different nanocomposites are given in Table 3. These data were chosen to conform to industrial settings.

For F_TA_, a significant change in mechanical behavior, characterized by an increase of stiffness at the origin, was evidenced. The ultimate tensile stress increased by 35%, and a slight increase of tensile strain (3%) was observed at 5 phr in comparison with F_R_. The moduli at 10% and 100% elongation gradually increased with POSS-A loading. At 20 phr, the modulus at 100% elongation increased by 116% compared to the compound without fillers, while the tensile at break decreased by 19.5% versus the 5 phr sample.

On the contrary, incorporation of POSS-P was found to have a negative impact on the mechanical properties of F_TP_ samples. The modulus at 10% of deformation remains relatively unaffected, whereas the modulus at 100%, tensile strength, and elongation at break decreased with increasing particle content (Figure 10). The observed decline relative to F_R_ suggests that micron-sized POSS domains that were observed on Figure 2 and Figure 3 to be due to the aggregation of nanofillers, may behave as weakening points during material deformation [30]. Since such micro-aggregates are also present in F_TA_ composites, their enhanced mechanical properties can be attributed to specific interactions between FKM polymer chains and POSS-A. This is consistent with the lowering of the temperature, at which the thermal initiation of exothermic reactions occurs (Figure 4a) due to the presence of reactive functional units at the surface of POSS-A particles, as discussed above, and this points toward a critical role of covalent grafting of POSS particles on FKM. In a similar study with one functional group on a POSS cage structure, Pan et al. [21] concluded that the increase of ultimate properties of a PDMS polymer was due to creation of links between the matrix and POSS. It is also worth mentioning here that the maximum tensile and elongation at break were obtained for F_TA_ at a 5 phr content, corresponding to the highest level of cross-linking according to DSC thermograms. Therefore, optimized resistance to high stress deformation may result from a trade-off between sufficient grafting and cross-linking density.

#### 3.4.2. DMA

**Figure 11:**
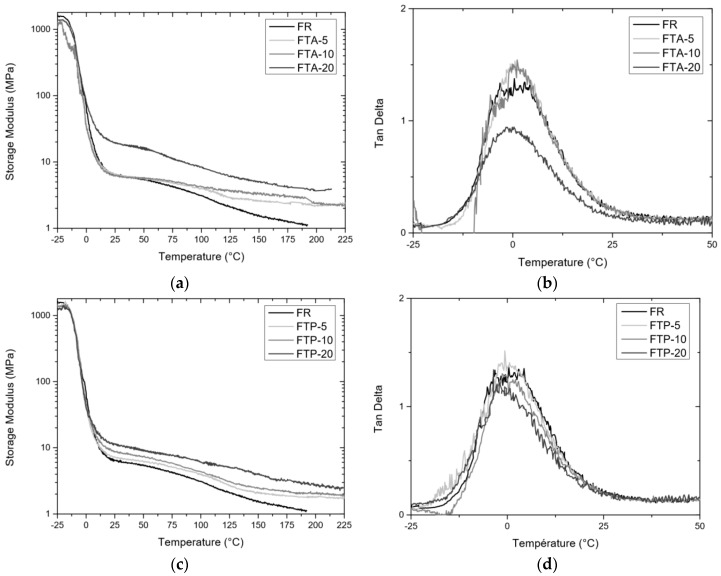
Temperature sweeps of the F_T_ nanocomposites: (**a**,**b**) F_TP_, (**c**,**d**) F_TA_.

Temperature sweeps realized by DMA (Figure 11) allow to determine the glass transition temperature and the rubbery state. Relaxation at high temperatures is associated with a change in the storage modulus, and occurs at a temperature higher to T_g_ as measured by DSC, as typically observed in literature [38]. The tan δ peak curve is commonly used to estimate the T_g_ [39]. The height (amplitude) of the tan δ curve is directly related to a material’s ability to dissipate energy through segmental motion, and the width of the peak informs the homogeneity of a system. Indeed, the height of tan δ peak is associated with the change in the magnitude of motion, as the amorphous polymer segments undergo a change from a glassy state to a rubbery state [40]. The pertinent parameters are summarized in Table 4.

From the obtained results, two main phenomena were observed here. First, POSS fillers impacted the behavior around the glass transition. A decrease of tan δ peak was noticed with increasing amount of POSS (Figure 11b,d). Systems with lower tan δ peaks had lower ratios of energy, absorbing viscous motions. The decrease in tan δ peak height may be caused by more restricted motion dynamics response due to the presence of POSS in the matrix [41]. These observations suggest that the chains movement was hindered by the presence of POSS. For F_TA_ and F_TP_, the Tg values were slightly shifted to higher temperatures in comparison with F_R_. However, a decrease of Tg for F_TA-20_ was noticed. This may be due to a lower grafting density because of higher POSS-A loading. POSS-A particles could indeed tend to react between themselves instead of grafting onto polymer chains during the vulcanization process.

Secondly, the evolution of storage modulus E’ in temperature showed a strong improvement upon POSS loading at high temperature (Figure 11a,c and Table 4). The phenomenon was more significant for F_TA_ composites. F_TA-20_ presented the highest increase of storage modulus E’ at 200 °C (+210% compared with F_R_ vs. +127% for F_TP-20_). Thus, the DMA characteristics suggested less motions of the macromolecular structure in the network and confirmed that POSS grafting could play a significant role. Formation of rigid fillers network usually provided additional levels of reinforcement. Filler–matrix and filler–filler interactions are unanimously associated with the decrease of the dynamic modulus with the strain amplitude, which is known as the Payne effect. This reinforcement has been highlighted in a previous study [42]. The enhanced values of E’ in the rubbery plateau region indicated that POSS-A may be a particularly suitable reinforcing filler in fluoroelastomers for high temperature applications.

## 4. Conclusions

Three POSS nanofillers have been incorporated in a FKM matrix. The nanocomposites were obtained as thin films. SEM/TEM and EDX imaging reveal a relatively proper dispersion state with the presence of micron-sized POSS domains that are smaller than aggregates that are attributed to the cross-linking system. The influence of POSS peripheric substituents and POSS content on thermal and thermomechanical properties has been studied. Although POSS fillers have little influence on the glass transition temperature, they are found to interfere with the vulcanization process. In particular, DSC measurements show that POSS-P hampers the cross-linking reaction, whereas the appearance of a new exothermic peak at lower temperature along with increased heat of reaction are indicative of the covalent grafting of POSS-A onto the FKM network. Both F_TA_ and F_TP_ composites exhibit improved thermal stability, with 50% weight loss temperature values reaching 493 °C and 502 °C for F_TA_ under air and nitrogen, respectively (+5 °C vs. the unfilled FKM matrix). Based on film morphology and thermal properties of the composites, POSS-A and POSS-P are selected as the most appropriate fillers. Incorporation of fillers leads to increased modulus. Consequently, the increase of stiffness leads to a decrease of mechanical response of F_TP_ composites because of the hindrance of chains motion induced by the presence of POSS particles. Yet, covalent grafting of POSS is found to play a critical role on the enhancement of mechanical properties of the composites. The maximum tensile and elongation at break are obtained for F_TA_ with 5 phr of filler and seem to be correlated to a trade-off between cross-linking and grafting density. Furthermore, storage modulus E’ is strongly enhanced in the rubbery plateau region up to 4.0 MPa at 200 °C with 20 phr loading (+210% compared with F_R_). Thus, POSS-A appears as a promising reinforcing agent in fluoroelastomers for high temperature applications.

Experiments are under way to further improve the dispersion prior to film deposition. To highlight a more pronounced impact of POSS ability to increase the thermal properties, tensile tests after thermal aging at high temperature and kinetics modelling of degradation are also in progress. Works are in progress to optimize the thermal and mechanical properties of composites FKM/POSS-A bearing various numbers of acrylate functional groups to study more deeply the effect of the grafting versus crosslinking reactions between POSS and FKM matrix.

## Figures and Tables

**Table 1:** Summary of T_g_ and ΔH of the composites (T_g_ and ΔH are reported in °C and J/g, respectively).

F_R_			phr	F_TA_			F_TP_			F_TF_		
Raw		Vulcanized	-	Raw		Vulcanized	Raw		Vulcanized	Raw		Vulcanized
T_g_	ΔH	T_g_		T_g_	ΔH	T_g_	T_g_	ΔH	T_g_	T_g_	ΔH	T_g_
−16.8	−24.6	−16.2	5	−18.8	−30.8	−15.6	−18.8	−20.5	−16.2	−16.4	−21.7	−14.4
			10	−21.0	−22.7	−16.5	−17.4	−24.1	−16.7	−22.6	−18.2	−14.6
			15	−19.7	−23.7	−14.8	−17.1	−15.6	−16.4	−20.5	−22.3	−15.5
			20	−16.1	−23.3	−16.5	−18.1	−13.7	−15.8	−19.7	−22.9	−15.9

**Table 2:** Summary of TGA results for POSS fillers and nanocomposites.

**phr**			**POSS-A**	**F_R_**	**F_TA_**			
				**0**	**5**	**10**	**20**	**40**
**Atmosphere**	**Nitrogen**	T_onset_ (°C)	371 ± 4	467 ± 2	469 ± 2	474 ± 3	478 ± 3	481 ± 1
		T_5%_ (°C)	321 ± 3	458 ± 1	450 ± 4	449 ± 2	419 ± 2	328 ± 4
		T_50%_ (°C)	432 ± 1	497 ± 1	497 ± 1	501 ± 1	502 ± 2	502 ± 1
		Ash (%)	3.8 ± 0.8	1.8 ± 0.2	2.2 ± 0.3	2.3 ± 0.02	3.8 ± 0.9	5.7 ± 0.9
		Theoretical ash (%)			0.2	0.3	0.6	1.1
	**Air**	T_onset_ (°C)	258 ± 6	456 ± 4	465 ± 1	467 ± 1	470 ± 2	474 ± 2
		T_5%_ (°C)	300 ± 1	430 ± 1	426 ± 1	421 ± 2	399 ± 1	336 ± 2
		T_50%_ (°C)	502 ± 1	488 ± 1	493 ± 1	493 ± 2	492 ± 2	493 ± 3
		Ash (%)	47.4 ± 0.9	2.0 ± 0.1	2.2 ± 0.08	2.4 ± 0.2	4.7 ± 0.4	12.3 ± 0.8
		Theoretical ash (%)			2.3	4.3	7.9	13.5
**phr**			**POSS-P**	**1**	**F_TP_**			
					**5**	**10**	**20**	**40**
**Atmosphere**	**Nitrogen**	T_onset_ (°C)	543 ± 6		465 ± 3	468 ± 3	468 ± 3	469 ± 1
		T_5%_ (°C)	518 ± 1		450 ± 3	452 ± 4	455 ± 3	452 ± 1
		T_50%_ (°C)	606 ± 1		495 ± 1	499 ± 1	501 ± 1	499 ± 2
		Ash (%)	74.7 ± 0.2		2.0 ± 0.1	2.1 ± 0.01	3.02 ± 0.9	3.5 ± 0.5
		Theoretical ash (%)			3.6	6.8	12.5	21.3
	**Air**	T_onset_ (°C)	518 ± 3		460 ± 2	459 ± 1	466 ± 2	463 ± 2
		T_5%_ (°C)	538 ± 1		428 ± 1	429 ± 3	428 ± 2	434 ± 1
		T_50%_ (°C)	583 ± 1		493 ± 1	495 ± 1	496 ± 1	494 ± 1
		Ash (%)	45.3 ± 0.2		1.5 ± 0.06	1.6 ± 0.1	2.4 ± 0.1	2.5 ± 0.2
		Theoretical ash (%)			2.2	4.1	7.6	12.9
**phr**			**POSS-F**		**F_TF_**			
						**10**	**20**	
**Atmosphere**	**Nitrogen**	T_onset_ (°C)	361 ± 6			467 ± 1	470 ± 1	
		T_5%_ (°C)	331 ± 2			354 ± 1	317 ± 2	
		T_50%_ (°C)	405 ± 6			497 ± 1	497 ± 1	
		Ash (%)	0.9 ± 0.1			1.7 ± 0.2	1.2 ± 0.3	
		Theoretical ash (%)				0.1	0.2	
	**Air**	T_onset_ (°C)	346 ± 6					
		T_5%_ (°C)	319 ± 1					
		T_50%_ (°C)	396 ± 6					
		Ash (%)	0.06 ± 0.3					
		Theoretical ash (%)						

**Table 3:** Mechanical parameters obtained from tensile tests at room temperature.

Sample	F_R_	F_TA_		F_TP_	
phr	0	5	20	5	20
Modulus at 10% elongation (MPa)	0.07 ± 0.03	0.2 ± 0.04	0.6 ± 0.1	0.07 ± 0.02	0.2 ± 0.03
Modulus at 100% elongation (MPa)	0.6 ± 0.05	0.9 ± 0.08	1.3 ± 0.09	0.4 ± 0.06	0.3 ± 0.02
Tensile at break (MPa)	2.4 ± 0.7	3.23 ± 0.4	2.6 ± 0.2	1.2 ± 0.1	0.5 ± 0.07
Elongation at break (%)	278.3 ± 45	286.0 ± 37	279.2 ± 25	243.4 ± 15	180.4 ± 14

**Table 4:** Mechanical parameters obtained from tensile tests at room temperature.

Sample	Temperature at the Peak of Tan δ T_g_ (°C)	Tan δ at Temperature of T_g_ (T = T_g_) Tan δ	Storage Modulus at 100 °C E’100 (MPa)	Storage Modulus at 200 °C E’200 (MPa)
F_R_	−2.8 ± 0.2	1.6 ± 0.09	3.05 ± 0.3	1.3 ± 0.4
F_TA-05_	1.4 ± 0.2	1.5 ± 0.07	4.0 ± 0.1	2.1 ± 0.07
F_TA-10_	1.5 ± 0.1	1.4 ± 0.2	4.8 ± 0.8	2.7 ± 0.6
F_TA-20_	0.2 ± 0.07	0.9 ± 0.01	9.2 ± 1.6	4.0 ± 0.6
F_TP-05_	−1.8 ± 0.09	1.5 ± 0.2	3.9 ± 0.1	1.8 ± 0.4
F_TP-10_	−0.9 ± 0.02	1.3 ± 0.07	4.4 ± 0.2	2.0 ± 0.7
F_TP-20_	1.1 ± 0.1	1.05 ± 0.03	5.6 ± 0.4	2.4 ± 0.2

## Supplementary Materials

The following are available online at http://www.mdpi.com/1996-1944/11/8/1358/s1, Figure S1: SEM micrographs of FKM polymer film (F), Figure S2: SEM micrographs of FR film, Figure S3: TEM micrographs of (a) POSS-A, (b) POSS-P, and (c) POSS-F nanoparticles, Figure S4: TEM micrographs of FKM polymer film (F), Figure S5: TEM micrographs of FR film, Figure S6: EDX spectrum of the black zone observed in Figure S5, for FR mixture, Figure S7: EDX spectrum of the black zone observed in Figure S5, for FR mixture, Figure S8: DSC curves of POSS nanofillers: (a) POSS-A, (b) POSS-P and (c) POSS-F, Figure S9: TGA curves of FKM polymer (F) under air and nitrogen, Figure S10: TGA curves of F_R_ under air and nitrogen.
