# Supplementary figures and images for: POSS Nanofiller-Induced Enhancement of the Thermomechanical Properties in a Fluoroelastomer Terpolymer
Source: Materials (Basel). 2018 Aug 6;11(8):1358. doi: 10.3390/ma11081358 (PMC6119982; doi:10.3390/ma11081358)

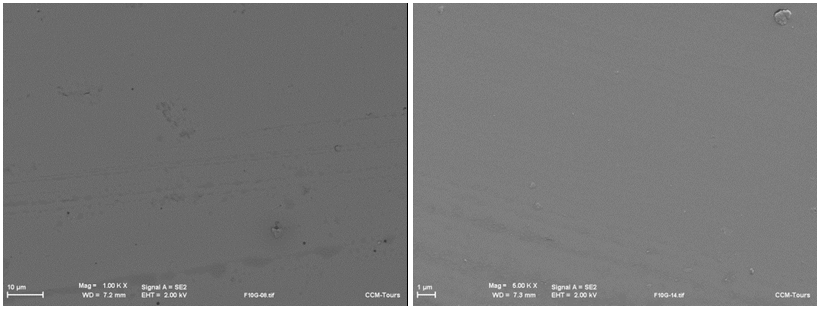

Supplement: Supplementary file 1 [file materials-11-01358-s001.zip › Supplementary Materials/Figure S1.PNG]

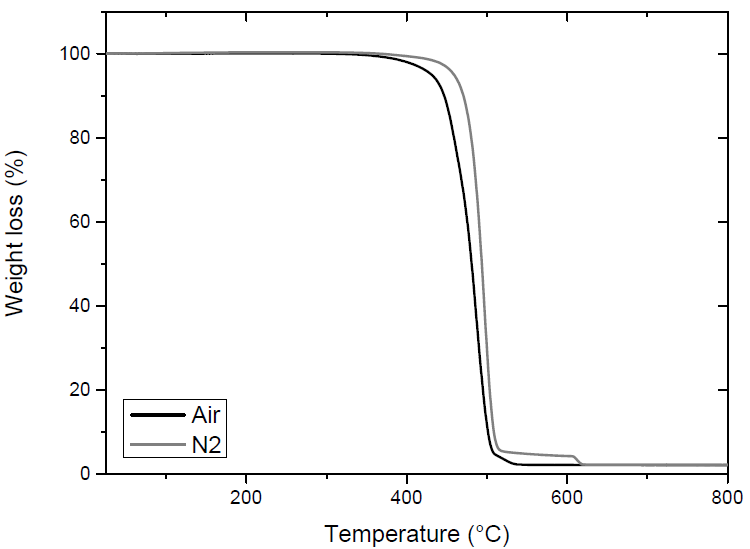

Supplement: Supplementary file 1 [file materials-11-01358-s001.zip › Supplementary Materials/Figure S10.PNG]

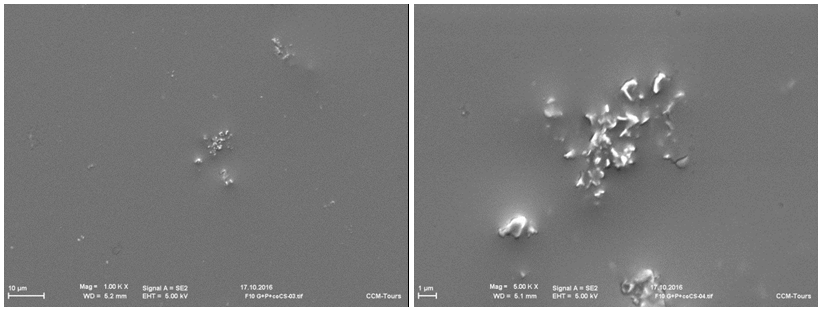

Supplement: Supplementary file 1 [file materials-11-01358-s001.zip › Supplementary Materials/Figure S2.PNG]

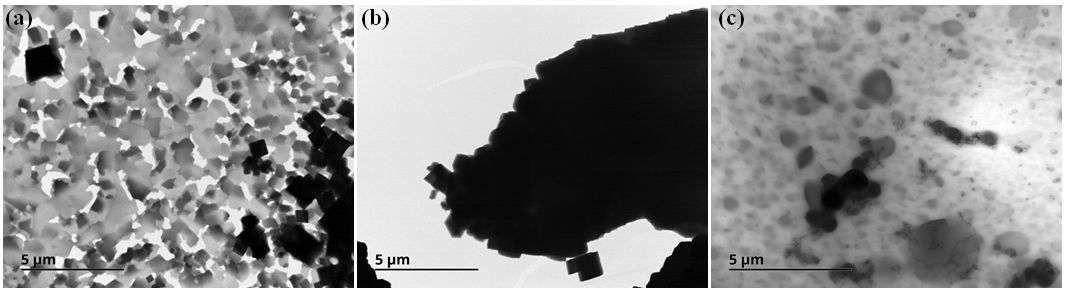

Supplement: Supplementary file 1 [file materials-11-01358-s001.zip › Supplementary Materials/Figure S3.PNG]

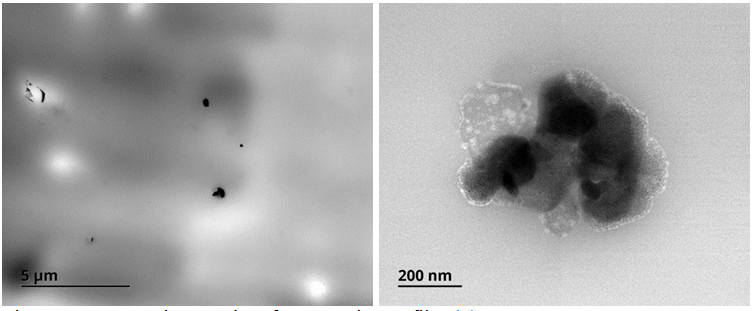

Supplement: Supplementary file 1 [file materials-11-01358-s001.zip › Supplementary Materials/Figure S4.PNG]

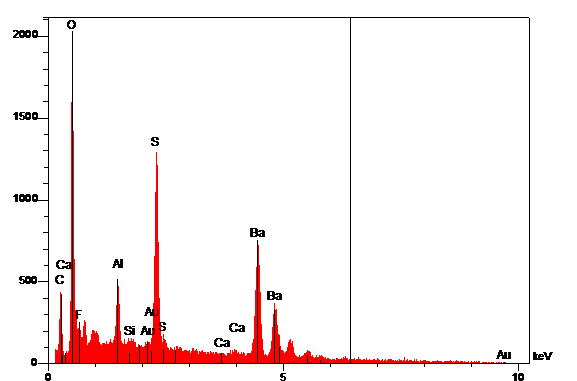

Supplement: Supplementary file 1 [file materials-11-01358-s001.zip › Supplementary Materials/Figure S6.PNG]

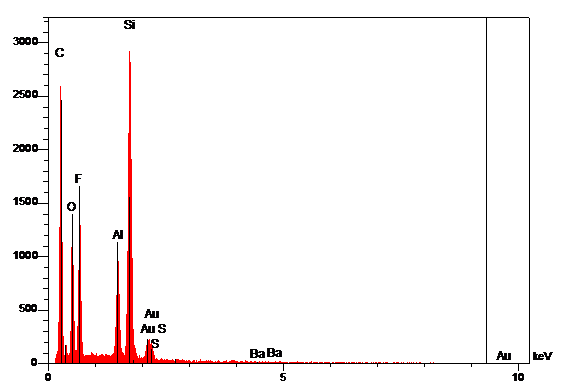

Supplement: Supplementary file 1 [file materials-11-01358-s001.zip › Supplementary Materials/Figure S7.PNG]

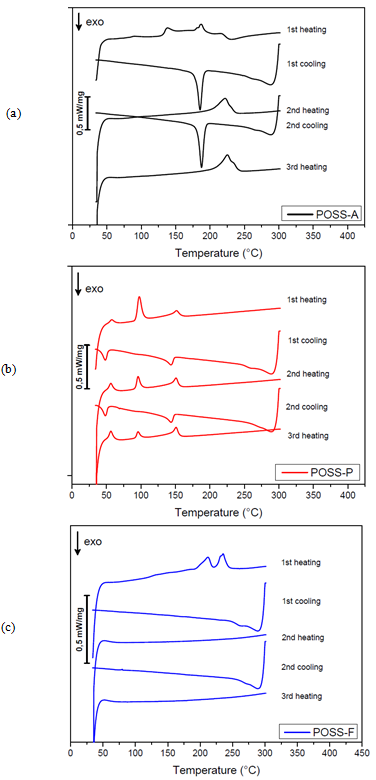

Supplement: Supplementary file 1 [file materials-11-01358-s001.zip › Supplementary Materials/Figure S8.PNG]

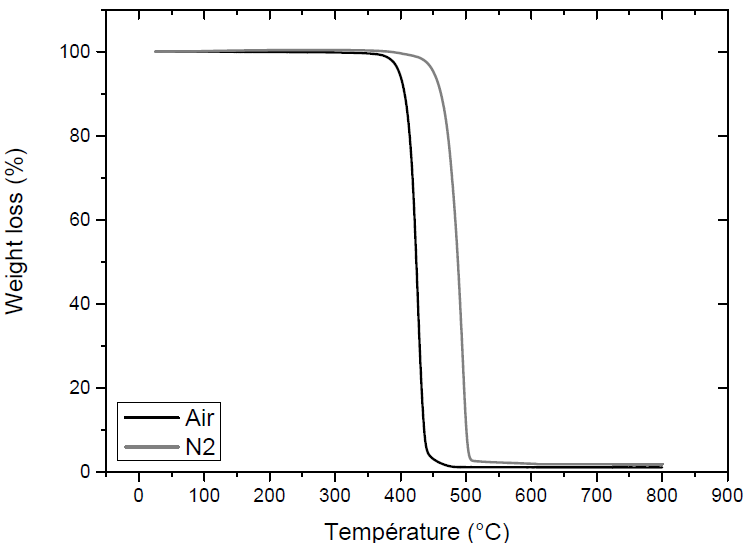

Supplement: Supplementary file 1 [file materials-11-01358-s001.zip › Supplementary Materials/Figure S9.PNG]
